# To Bridge or Not to Bridge? A Meta‐Analysis of Intravenous Thrombolysis Before Thrombectomy in Large Ischemic Core Strokes

**DOI:** 10.1002/brb3.71052

**Published:** 2025-11-21

**Authors:** Abdelrahman Elgharabawi, Mostafa Hossam El Din Moawad, Reham M. Wagih, Yousr Ahmed, Mohammed Elkholy, Ibrahim Serag, Ibraheem M. Alkhawaldeh, Mahmoud Elsayed, Abdelrahman Elkholy, Ahmed Abdelraouf, Obai Yousef, Younes Nabgouri, Mohamed Abouzid

**Affiliations:** ^1^ Faculty of Biology, Medicine and Health Manchester University Manchester The United Kingdom; ^2^ Alexandria Main University Hospital Alexandria Egypt; ^3^ Faculty of Medicine Suez Canal University Ismailia Egypt; ^4^ Department of Total Parenteral Nutrition Alexandria Main University Hospital Alexandria Egypt; ^5^ Department of Pulmonology and Critical Care Johns Hopkins University Baltimore Maryland USA; ^6^ The Laboratory for Minimally Invasive Tumor Therapies, Department of Radiology Beth Israel Deaconess Medical Center/Harvard Medical School Boston Massachusetts USA; ^7^ Faculty of Medicine Mansoura University Mansoura Egypt; ^8^ Faculty of Medicine Mutah University Al‐Karak Jordan; ^9^ Stroke and Neurovascular Regulation Laboratory Charlestown Massachusetts USA; ^10^ Faculty of Medicine Alexandria University Alexandria Egypt; ^11^ Faculty of Medicine Al‐Azhar University Cairo Egypt; ^12^ Department of Neurosurgery Tishreen University Hospital Latakia Syria; ^13^ Department of Internal Medicine First Pavlov State Medical University Saint Petersburg Russia; ^14^ Department of Physical Pharmacy and Pharmacokinetics, Faculty of Pharmacy Poznan University of Medical Sciences Poznan Poland; ^15^ Doctoral School Poznan University of Medical Sciences Poznan Poland

**Keywords:** bridging, endovascular thrombectomy, intravenous thrombolysis, large ischemic core, stroke

## Abstract

**Background:**

Endovascular thrombectomy (EVT) is the standard treatment for acute ischemic stroke (AIS) caused by large vessel occlusion (LVO). However, the role of preceding intravenous thrombolysis (IVT) in patients with sizable ischemic core infarcts remains unclear. This systematic review and meta‐analysis aimed to compare the clinical efficacy and safety of bridging therapy (IVT followed by EVT) versus EVT alone in this specific high‐risk subgroup.

**Methods:**

Following PRISMA guidelines, a comprehensive literature search was conducted across PubMed, Web of Science, and Scopus to identify studies comparing bridging therapy (IVT + EVT) with EVT alone in patients with large ischemic cores. Primary efficacy outcomes included favorable functional status, defined as modified Rankin Scale (mRS) scores of 0–1 and 0–2 at follow‐up. Primary safety outcomes were rates of symptomatic intracranial hemorrhage (sICH) and any intracranial hemorrhage (ICH). Secondary outcomes assessed successful reperfusion and mortality. Data were pooled using random‐effects models and reported as risk ratios (RR) with 95% confidence intervals (CI).

**Results:**

Seven cohort studies met the inclusion criteria. No significant differences were observed between the two treatment strategies in achieving mRS 0–1 (*RR* = 0.78; 95% CI: 0.52–1.19; *p* = 0.25) or mRS 0–2 (*RR* = 0.70; 95% CI: 0.46–1.08; *p* = 0.11). Similarly, rates of sICH (*RR* = 0.93; 95% CI: 0.67–1.28; *p* = 0.64), any ICH (*RR* = 0.90; 95% CI: 0.79–1.04; *p* = 0.15), successful recanalization (*RR* = 0.92; 95% CI: 0.83–1.03; *p* = 0.14), and mortality (*RR* = 1.08; 95% CI: 0.96–1.21; *p* = 0.20) were comparable between groups.

**Conclusion:**

In patients with large ischemic core infarcts, administering IVT prior to EVT does not confer significant clinical or procedural advantages over EVT alone. These findings underscore the need for further randomized controlled trials to inform optimal treatment approaches for this challenging patient population.

## Graphical abstract

Based on the included 7 studies, in patients with large ischemic core infarcts, IVT before EVT offers no significant clinical or procedural benefit compared to EVT alone.

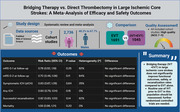

## 1 Introduction

Stroke is a sudden neurological deficit caused by interrupted cerebral blood flow, either from vessel blockage (ischemic) or rupture (hemorrhagic), and remains a leading cause of death and disability worldwide. Ischemic stroke often results from atherosclerosis, cardioembolism, or small vessel disease, while hemorrhagic stroke is linked to hypertension and vascular malformations. Risk factors include modifiable ones—such as hypertension, diabetes, smoking, and obesity—and non‐modifiable ones like age, sex, and genetics. Early detection and control of these risks are essential for prevention and improved outcomes (Berkhemer et al. 2015; Saver et al. 2015; Jovin et al. 2015). Between 2015 and 2018, seven pivotal randomized controlled trials (RCTs) firmly established endovascular thrombectomy (EVT) as the gold‐standard treatment for acute ischemic stroke (AIS) caused by large vessel occlusion (LVO) (Berkhemer et al. 2015; Saver et al. 2015; Jovin et al. 2015; Goyal et al. 2015; Campbell et al. 2015; Nogueira et al. 2018; Albers et al. 2018; Powers et al. 2019). More recently, advancements in endovascular techniques and the expansion of clinical eligibility criteria have led to the publication of four additional trials supporting EVT in patients with extensive infarct cores—commonly defined by an Alberta Stroke Program Early CT Score (ASPECTS) of ≤ 5 (Sarraj et al. 2023; Huo et al. 2023; Yoshimura et al. 2022; Bendszus et al. 2023). These individuals were largely excluded from earlier studies due to concerns over limited salvageable brain tissue and an elevated risk of intracranial hemorrhage (ICH) (Goyal et al. 2016). In this context, the value of intravenous thrombolysis (IVT) administered prior to EVT remains a matter of ongoing debate.

Current standard care for AIS within 4.5 h of symptom onset typically includes IVT with alteplase, regardless of the presence of early ischemic changes on imaging (Lees et al. 2016; Thomalla et al. 2018; Ma et al. 2019; Broocks et al. 2020). While the efficacy of alteplase in improving outcomes for AIS has been demonstrated in several landmark trials (Hacke et al. 2008; Wahlgren et al. 2008), its benefit in patients with large ischemic cores who are candidates for EVT is far less certain. The American Heart Association (AHA) guidelines currently recommend IVT for patients with mild to moderate early ischemic changes on non‐contrast CT (NECT), but discourage its use in the presence of extensive hypoattenuation, reflecting a lack of robust data for this subgroup (Powers et al. 2019; Broocks et al. 2020).

As randomized trials begin to include patients with larger core infarcts, reevaluating the role of IVT in bridging therapy becomes increasingly relevant. While some clinicians advocate for the continued use of IVT prior to EVT, others argue that it may increase the risk of hemorrhagic complications without providing substantial additional benefit—especially in cases of already extensive infarction. This ongoing uncertainty underscores the need for a clearer understanding of whether bridging therapy (IVT followed by EVT) offers superior clinical outcomes compared to EVT alone in patients with large ischemic cores.

To address this knowledge gap, we conducted a systematic review and meta‐analysis aimed at comparing the safety and efficacy of IVT plus EVT versus EVT alone in patients presenting with extensive core infarcts. The findings aim to inform clinical decision‐making and guide future research into optimal revascularization strategies for this high‐risk population.

## 2 Methods

This systematic review and meta‐analysis was conducted in accordance with the PRISMA (Preferred Reporting Items for Systematic Reviews and Meta‐Analyses) guidelines (Moher et al. 2009). A comprehensive literature search was performed across PubMed, Web of Science, and Scopus, covering all studies published from inception until February 2025. The search strategy incorporated key terms and corresponding MeSH headings, including *“Intravenous thrombolysis,” “Alteplase,” “Tissue plasminogen activator,” “Tenecteplase,” and “Bridging,”* combined with *“Mechanical thrombectomy” OR “Endovascular thrombectomy,”* and *“Large core,” “Large ischemic core,” “Large infarct,” or “Large ischemic infarct.”*

### 2.1 Study Selection and Eligibility Criteria

All retrieved references were imported into Rayyan for systematic screening (Johnson and Phillips 2018). Titles and abstracts were initially reviewed for relevance, followed by full‐text evaluation based on pre‐specified eligibility criteria. Included studies involved adult patients with AIS due to large infarct cores, comparing outcomes between EVT alone and bridging therapy (IVT followed by EVT). The primary efficacy outcomes were functional independence, defined as modified Rankin Scale (mRS) scores of 0–1 and 0–2. Primary safety outcomes included rates of symptomatic intracranial hemorrhage (sICH) and any form of intracranial hemorrhage (ICH). Secondary outcomes were successful reperfusion (efficacy) and mortality (safety). Eligible study designs included RCTs and observational studies (cohort and case‐control), while case reports, editorials, and reviews were excluded.

### 2.2 Data Extraction

Two reviewers independently extracted data into structured Microsoft Excel spreadsheets. Extracted variables included study design, sample size, mean age, sex distribution, baseline stroke severity measured by the National Institutes of Health Stroke Scale (NIHSS), and extent of early ischemic changes as assessed by the Alberta Stroke Program Early CT Score (ASPECTS). Outcome measures for both treatment arms were recorded, specifically the number of patients achieving mRS 0–1 and 0–2, as well as incidences of sICH, any ICH, successful recanalization, and all‐cause mortality.

### 2.3 Quality Assessment

The methodological quality of included observational studies was appraised using the Newcastle‐Ottawa Scale (NOS) (Wells et al. 2014). This scale evaluates three domains—selection, comparability, and outcome—and assigns a total score ranging from 0 to 9 stars. Studies receiving 7–9 stars were considered high quality, 4–6 stars moderate quality, and 0–3 stars low quality. Two independent authors performed the assessments, with discrepancies resolved by consensus or consultation with a third reviewer.

### 2.4 Statistical Analysis

Meta‐analyses were performed using Review Manager (RevMan) software (Deeks and Higgins 2010). Risk ratios (RRs) with 95% confidence intervals (CIs) were calculated for dichotomous outcomes. Heterogeneity across studies was evaluated using the I^2^ statistic, with values of 25%, 50%, and 75% indicating low, moderate, and high heterogeneity, respectively. A *p*‐value of ≤ 0.05 was considered statistically significant. Random‐effects models were applied to account for potential heterogeneity. Sensitivity analyses were conducted using a leave‐one‐out approach to evaluate the robustness of pooled estimates.

## 3 Results

### 3.1 Searching and Screening

**FIGURE 1:**
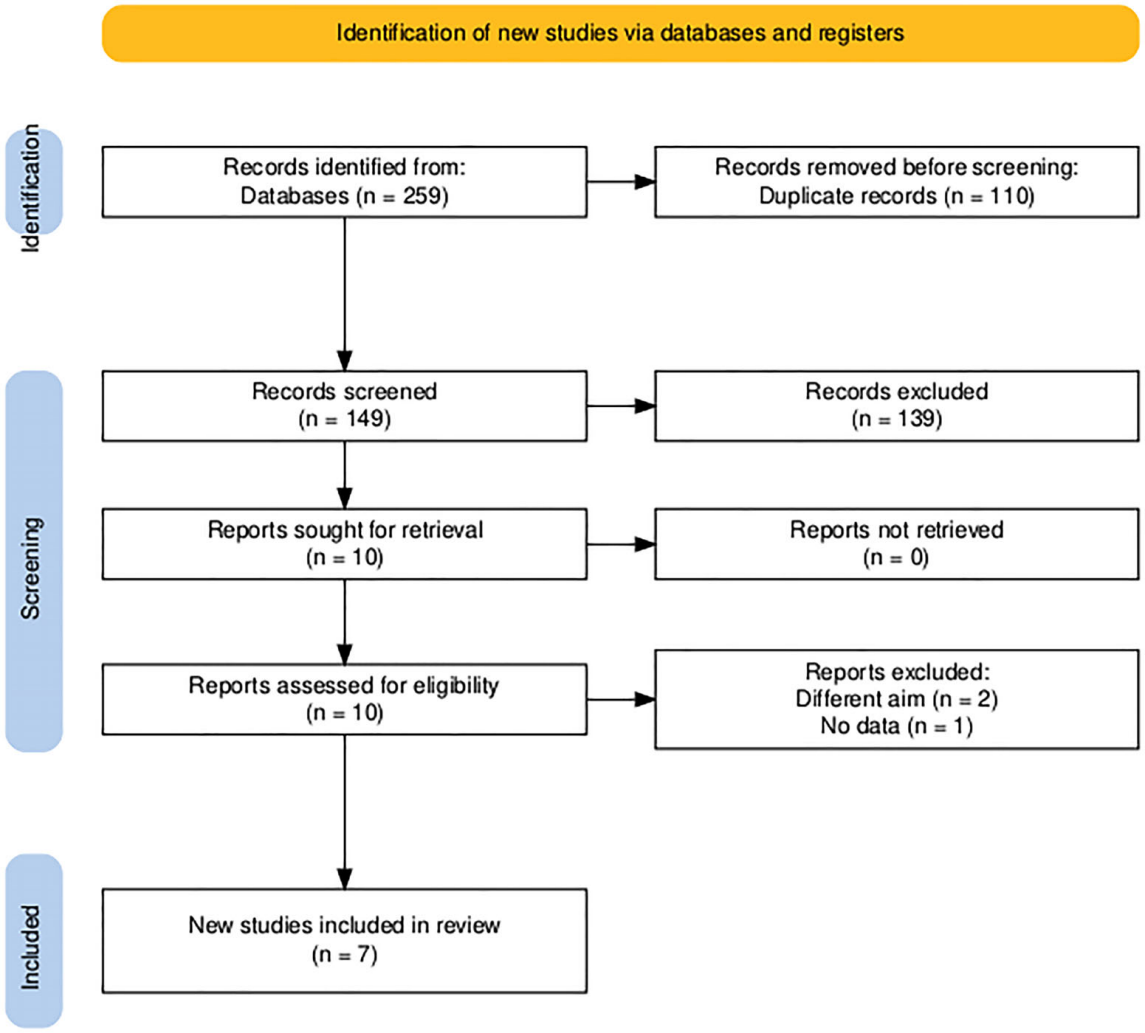
PRISMA flow chart of the screening process.

The search strategy identified a total of 259 records, out of which 110 were duplicates and removed. Following a review of the titles and abstracts of the remaining 149 articles, 10 studies met the criteria for a full‐text assessment. Ultimately, seven articles were found to meet the eligibility criteria and were included in the final systematic review and meta‐analysis (Figure 1) (Anadani et al. 2023; Sun et al. 2025; Jia et al. 2022; Yuan et al. 2025; Broocks et al. 2023; Derraz et al. 2023; Shindo et al. 2024).

### 3.2 Quality Assessment

According to NOS, all the included cohort studies were considered of high quality (Table 1).

**TABLE 1:** Quality assessment of cohort studies using the Newcastle Ottawa Scale tool.

Study name	The level of representation of the affected cohort (★)	Identification of the unexposed cohort (★)	Determination of exposure (★)	Evidence that the outcome of interest was absent at the commencement of the research (★)	Comparison of cohorts based on design or assessment (max ★★)	Was the follow‐up duration sufficient for consequences to manifest? (★)	Evaluation of results (★)	Assessment of cohort follow‐up sufficiency (★)	Quality level
Sun 2025	★	★	★	★	★★	★	★	★	High
Yuan 2024	★	★	★	★	★★	★	★	★	High
Broocks 2023	★	—	★	★	★★	★	★	★	High
Jia 2022	★	★	★	★	★★	—	★	★	High
Derraz 2023	★	—	★	★	★★	★	★	★	High
Shindo 2023	★	★	★	★	★★	★	★	★	High
Anadani 2023	★	★	★	★	★★	★	★	★	High

### 3.3 Baseline Characteristics

Seven cohort studies were included in the current analysis. Across the studies, sample sizes varied significantly, with the largest cohort comprising 716 EVT and 587 bridging therapy patients (Derraz et al. 2023) and the smallest cohort including 25 EVT and 31 bridging therapy patients (Jia et al. 2022). The mean (standard deviation [SD]) age of patients was relatively consistent across studies, generally ranging from 67 (15.3) to 76.3 (11.6) years. The proportion of male patients varied from approximately 48.2% to 67.7%. Stroke severity, as assessed by the NIHSS, had a median (interquartile range [IQR]) ranging from 17 (14–20) to 22 (17–26) across studies, reflecting a broad spectrum of baseline neurological deficits. The ASPECTS, used to quantify the extent of early ischemic changes, showed slight variation between studies, ranging from 0 to 5. (Table 2)

**TABLE 2:** Baseline characteristics of the included studies.

Study ID	Study design	Sample size		Age, mean (SD)		Male, n (%)		NIHSS, median (IQR)		ASPECTS, median (IQR)	
		EVT alone	Bridging	EVT alone	Bridging	EVT alone	Bridging	EVT alone	Bridging	EVT alone	Bridging
Sun 2025	Cohort	368	122	68.3 (14.9)	69 (12)	209 (56.8)	72 (59)	17 (14–21)	17 (14–20)	4 (2–5)	4 (2–5)
Yuan 2024	Cohort	77	45	67.5 (7.7)	67.1 (7.8)	39 (50.6)	27 (60)	17 (14–20)	18 (13–20)	NR	NR
Broocks 2023	Cohort	78	90	NR	NR	NR	NR	19 (16–22)		4 (3‐5)	
Jia 2022	Cohort	25	31	70.2 (11.8)		27 (48.2)		20 (15– 23)		0‐4	
Derraz 2023	Cohort	716	587	68 (15.4)	67 (15.3)	369 (51.5)	340 (57.9)	19 (16–23)	19 (16–22)	4 (3–5)	4 (3–5)
Shindo 2023	Cohort	74	26	76.1 (9.5)	76.3 (11.6)	NR	NR	22 (17–26)	21 (18–26)	3 (3–4)	3 (3–4)
Anadani 2023	Cohort	353	144	67.7 (14.8)	67.1 (13.8)	239 (67.7)	74 (51.4)	18.9 (6.3)*	18.4 (5.9)*	5 (1)*	5 (1)*

### Abbreviations: EVT, Endovascular Thrombectomy; IQR, Interquartile Range; NR, Not Reported; SD, Standard Deviation

### *Data Reported as Mean and SD

### 3.4 Statistical Analysis

**FIGURE 2:**
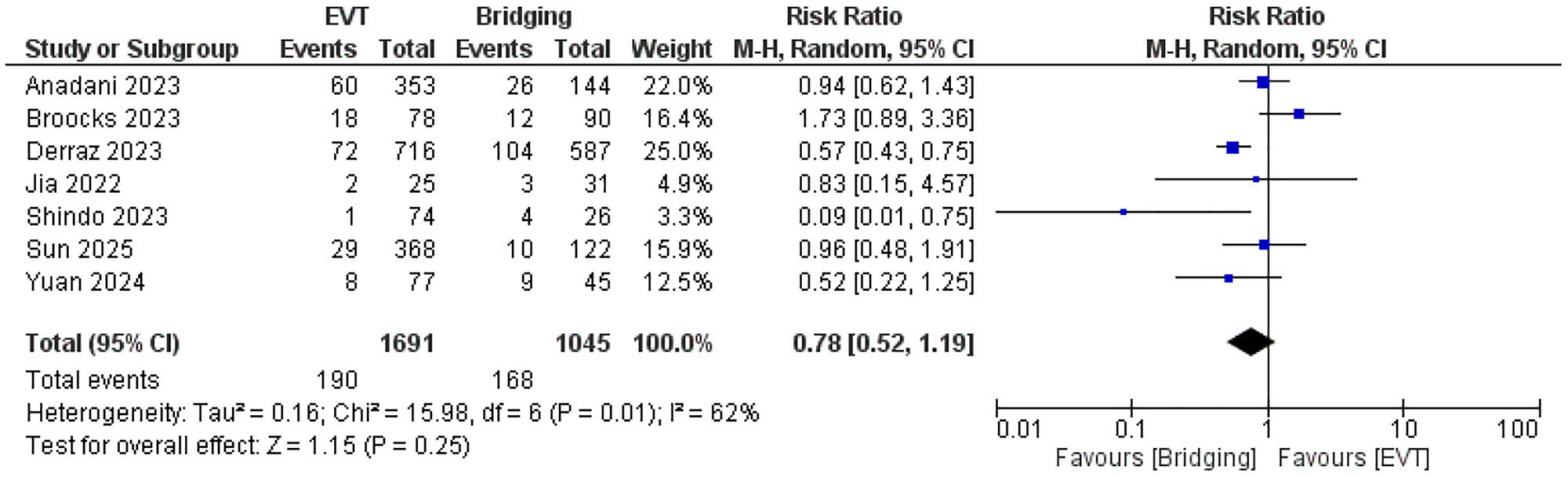
Comparison between endovascular thrombectomy and bridging therapy in mRS 0–1.

No significant difference was Figure 1 observed between EVT and bridging therapy regarding the incidence of mRS 0–1 with *RR* = 0.78 (95% CI: 0.52, 1.19, *p* = 0.25), and *I* ^2^ = 62%, *p* = 0.01, showing significant heterogeneity (Figure 2) that was resolved after leave‐one‐out analysis by removing Broocks et al. (2022) study, and this also favored bridging therapy compared with EVT with *RR* = 0.68 (95% CI: 0.48, 0.98, *p* = 0.04) and *I* ^2^ = 43%, *p* = 0.12. (Supplementary figure 1).

**FIGURE 3:**
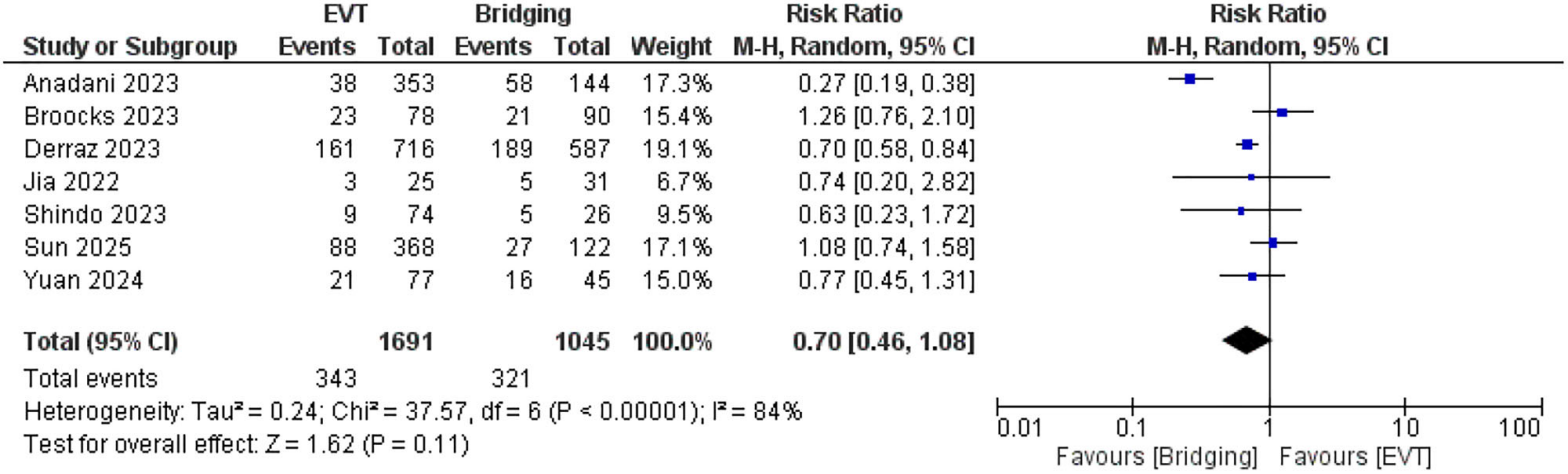
Comparison between endovascular thrombectomy and bridging therapy in mRS 0–2.

Also, no significant difference was observed between EVT and bridging therapy regarding the incidence of mRS 0–2 with *RR* = 0.7 (95% CI: 0.46, 1.08, *p* = 0.11), and *I* ^2^ = 84%, *p* < 0.00001. (Figure 3) This heterogeneity was resolved using leave‐one‐out analysis by removing the Anadani et al. (2023) study, as *I* ^2^ was 37%, *p* = 0.16. (Supplementary figure 2).

**FIGURE 4:**
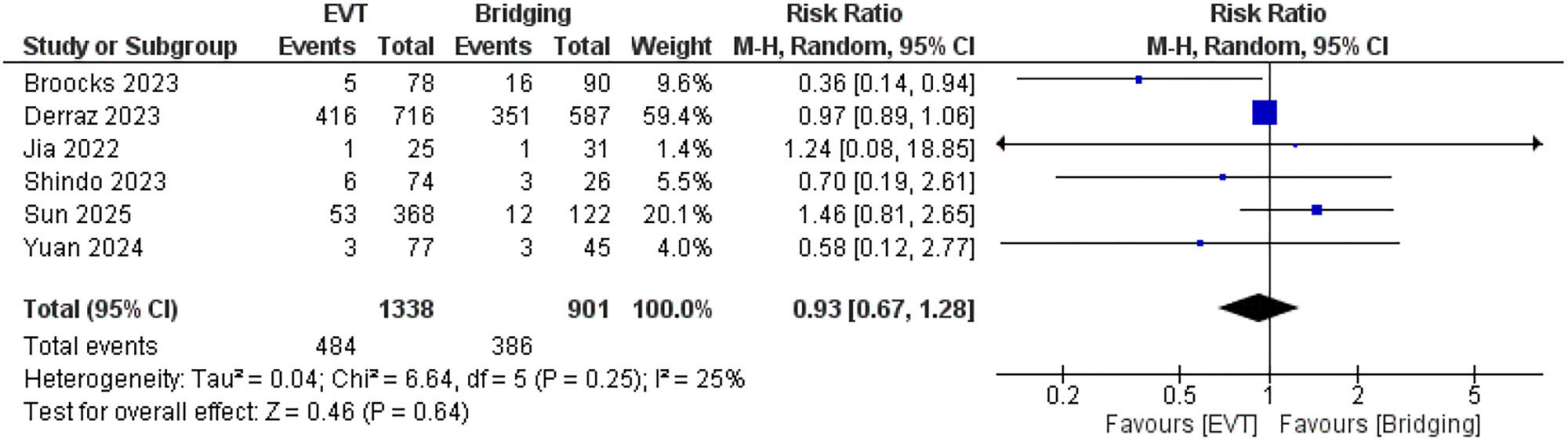
Comparison between endovascular thrombectomy and bridging therapy in the risk of sICH.

**FIGURE 5:**
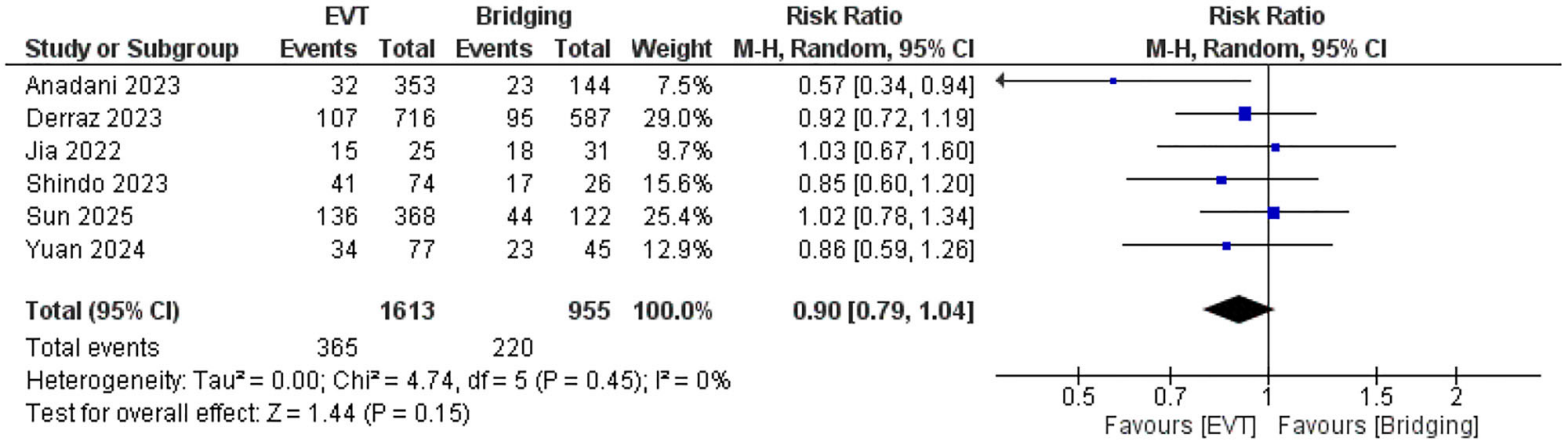
Comparison between endovascular thrombectomy and bridging therapy in the risk of ICH.

The pooled analysis demonstrated no significant difference between EVT and bridging therapy in the risk of sICH (*RR*: 0.93; 95% CI: 0.67, 1.28, *p* = 0.64), and *I* ^2^ = 25%, *p* = 0.25 (Figure 4) and the risk of ICH (*RR*: 0.9; 95% CI: 0.79, 1.04, *p* = 0.15), and *I* ^2^ = 0%. (Figure 5)

**FIGURE 6:**
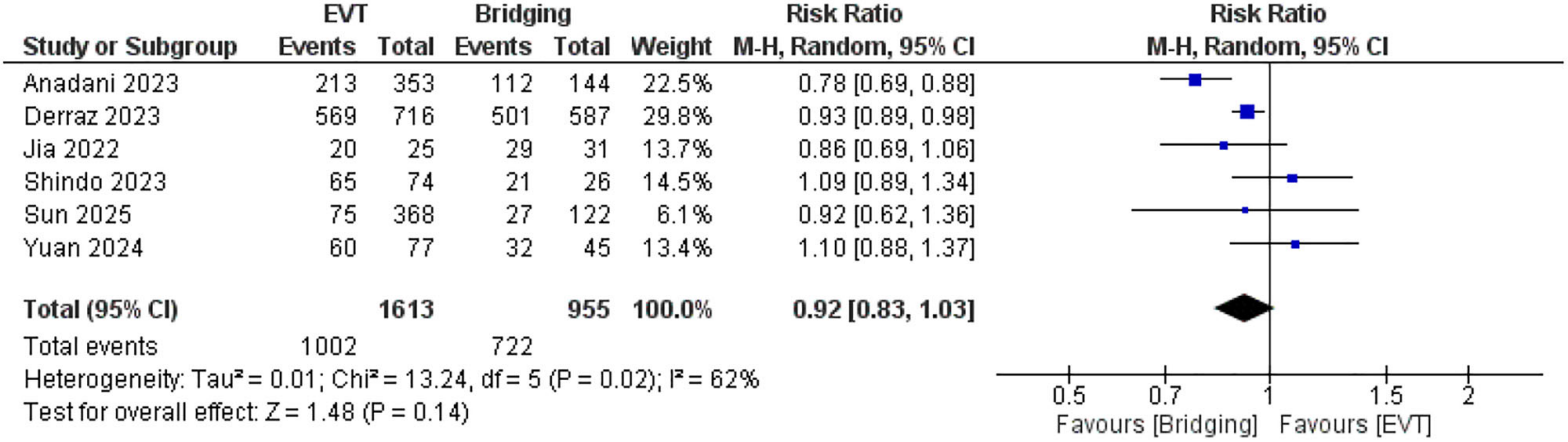
Comparison between endovascular thrombectomy and bridging therapy in successful recanalization.

**FIGURE 7:**
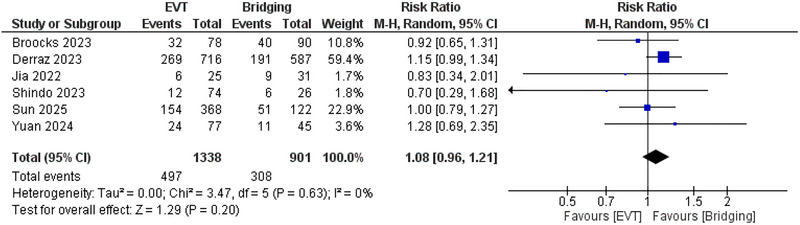
Comparison between endovascular thrombectomy and bridging therapy in the risk of mortality.

Moreover, no significant difference was observed between EVT and bridging therapy regarding successful recanalization (*RR*: 0.92; 95% CI: 0.83, 1.03, *p* = 0.14), and *I* ^2^ = 62%, *p* = 0.02. (Figure 6) This heterogeneity was resolved after leave‐one‐out of the Anadani et al. (2023) study as *I* ^2^ = 14%, *p* = 0.32. (Supplementary figure 3) Furthermore, no significant difference was observed regarding the risk of mortality (*RR*: 1.08; 95% CI: 0.96, 1.21, *p* = 0.2), and *I* ^2^ = 0%. (Figure 7).

## 4 Discussion

This systematic review and meta‐analysis assessed whether administering intravenous thrombolysis (IVT) before endovascular thrombectomy (EVT) confers additional benefit or risk in AIS patients with large infarct cores. Our findings revealed no significant differences between bridging therapy (IVT + EVT) and EVT alone in terms of functional outcomes (mRS 0–1, mRS 0–2), intracranial hemorrhage risk, successful reperfusion rates, or mortality. These results suggest that IVT may not offer meaningful clinical or safety advantages for this high‐risk subgroup.

Historically, IVT has been associated with improved outcomes in stroke management, though at the cost of increased bleeding risk—particularly symptomatic intracranial hemorrhage (sICH) (Smith et al. 2022). Several pathophysiological mechanisms may explain the limited benefit of IVT in large core infarcts. Large infarcts are often associated with severe and irreversible ischemic injury, extensive cytotoxic edema, and early disruption of the blood–brain barrier. This structural compromise facilitates the extravasation of blood components into the brain parenchyma once reperfusion occurs, significantly increasing the likelihood of hemorrhagic transformation. Moreover, in the setting of a large ischemic core, microvascular integrity is markedly impaired due to endothelial injury, inflammatory cell infiltration, and activation of matrix metalloproteinases, which degrade the extracellular matrix and further weaken the blood–brain barrier. The “no‐reflow phenomenon,” characterized by microvascular obstruction despite recanalization, may also limit the delivery of oxygen and nutrients to already damaged tissue, reducing the potential for functional recovery. Additionally, reperfusion in these fragile vascular beds can exacerbate oxidative stress and excitotoxicity, compounding tissue damage and increasing the risk of symptomatic intracranial hemorrhage. Mechanistically, alteplase may impair vascular integrity by promoting matrix metalloproteinase activity in endothelial cells and triggering immune‐mediated disruption of the blood–brain barrier (Wang et al. 2003; Shi et al. 2021). In patients with large infarcts, this may significantly elevate the risk of hemorrhagic transformation. Indeed, prior studies have reported nearly 50% mortality among those who develop sICH following IVT in the context of large core infarcts (Álvarez‐Sabín et al. 2013). These concerns warrant careful risk‐benefit evaluation when considering IVT in such cases.

One of the central challenges in managing patients with large infarcts is the elevated risk of hemorrhage following reperfusion therapy (Campbell et al. 2022). Existing literature remains inconclusive on the value of IVT in this population. For example, post‐hoc data from the DIRECT‐MT trial did not demonstrate a clear effect of IVT among patients with ASPECTS < 5, though small subgroup sizes limited statistical power (Broocks et al. 2020). Other observational studies suggested that IVT may be more effective in patients with moderate ASPECTS scores (6–10), while offering limited or no benefit in those with lower scores (Broocks et al. 2022). Similarly, analyses involving patients with extensive infarcts have linked IVT to worse functional outcomes and greater bleeding risk (Broocks et al. 2023). Yet, findings from studies such as Anadani et al. (2023), which focused on homogenous low‐ASPECTS populations, did not show an increase in hemorrhagic events with bridging therapy, further illustrating the heterogeneity across studies.

Another layer of complexity is introduced by the imaging modality used to define infarct size. CT‐based ASPECTS tends to be less sensitive than MRI in detecting early ischemic changes, and poor agreement between CT and MRI‐derived ASPECTS may account for inconsistent outcome predictions (Anadani et al. 2023; Meyer et al. 2021; McTaggart et al. 2015; Bracard et al. 2016; Román et al. 2018; Kaesmacher et al. 2019). Notably, EVT has shown benefit in patients selected with MRI‐based low ASPECTS, while CT‐based selection has yielded less promising results—underscoring the importance of imaging modality in treatment decision‐making.

The impact of IVT may also vary depending on procedural factors and patient characteristics. A post‐hoc analysis of the MR CLEAN NO‐IV trial found that IVT improved outcomes only in patients treated with contact aspiration, but not with stent retrievers (Rinkel et al. 2022). However, a separate analysis by Anadani et al. (2023) did not identify a significant interaction between the type of thrombectomy device and IVT in patients with large core strokes. Additionally, comorbidities such as atrial fibrillation may influence IVT outcomes. While one study using the STAR registry linked IVT use to an increased risk of bleeding in patients with atrial fibrillation (Akbik et al. 2022), other multicenter studies have not replicated this association (Mujanovic et al. 2022).

Altogether, these findings highlight the uncertainty and variability surrounding the role of bridging therapy in patients with large ischemic cores. Our meta‐analysis, encompassing a broader dataset, adds to the growing body of evidence suggesting that IVT may not offer added benefit in this specific population. Nonetheless, further high‐quality randomized trials are warranted to clarify patient subgroups that may derive benefit—or harm—from IVT prior to EVT.

The current AHA guidelines propose the administration of alteplase in cases with early ischemia alterations on CT of mild to moderate severity. Nonetheless, IVT is contraindicated in patients exhibiting significant areas of clear hypoattenuation (Powers et al. 2018). This underscores a dual issue: (1) How can early hypoattenuation of moderate extent be reliably differentiated from pronounced hypoattenuation in the absence of a definitive threshold? (2) What is the extent of the influence of the recognized poor inter‐rater reliability of early ischemic changes, and does this consequently result in significant variations in the decision‐making process regarding IVT in patients with low ASPECTS in routine clinical practice? (Schröder and Thomalla 2016; von Kummer et al. 1997) The ambiguous influence of IVT on patients with low ASPECTS may significantly impact the results of ongoing EVT trials in this population, potentially leading to the failure of these studies. Consequently, the uniform implementation of IVT in patients with low ASPECTS warrants additional assessment, especially considering the emergence of improved therapy selection instruments (McDonough et al. 2021). The scoring of ASPECTS could be enhanced with the implementation of standardized automated technologies, which are already accessible and demonstrate accuracy in predicting the actual ultimate infarct volume, in contrast to subjective ASPECTS assessments (Austein et al. 2019; Kuang et al. 2019; Sundaram et al. 2019). Secondly, objective quantitative metrics could enhance ASPECTS, such as quantitative assessment of lesion water absorption (Nawabi et al. 2019; Minnerup et al. 2016; Broocks et al. 2018). It is essential to recognize that the ASPECTS rating is founded on binary subjective evaluation criteria (hypoattenuation present/absent). Consequently, it does not provide additional quantification of the extent of hypoattenuation. Early brain tissue infarction is characterized by net water absorption, which correlates directly with lesion hypodensity and a rise in volume (i.e., extracellular edema). The physics underlying the reduction of CT attenuation in ischemic tissue necessitates a net influx of water (i.e., edema), as demonstrated in prior in vitro and in vivo studies (Broocks et al. 2018). Consequently, this further quantitative indicator could enhance the interpretation of existing guidelines by more precisely distinguishing early ischemic hypoattenuation from pronounced hypodensity, thereby improving the selection of patients with low ASPECTS for IVT delivery. Moreover, the precise extent of hypoattenuation may serve as a predictor for the responsiveness to IVT in individuals with low ASPECTS or function as a mechanism for early risk assessment of sICH (Nawabi et al. 2019; Nawabi et al. 2021).

The analysis included only observational studies, introducing potential biases compared with RCTs; however, most of them were prospective, which strengthens the study compared with retrospective ones. Also, the inadequate study of the effect of various factors such as imaging modalities, timing of treatment, associated comorbidities, and types of EVT is considered a limitation. Large‐scale RCTs are needed to provide definitive evidence regarding the efficacy and safety of IVT before EVT in patients with extensive ischemic core infarcts. Future RCTs investigating IVT in large core infarcts should incorporate strict, standardized patient selection criteria to reduce heterogeneity. Imaging protocols should rely on objective, automated ASPECTS scoring or quantitative lesion water uptake analysis to minimize inter‐rater variability and improve infarct core characterization. MRI diffusion‐weighted imaging or CT perfusion could be used to confirm core size and assess collateral circulation quality, ensuring that patients with at least partial collateral flow are included. Trials should stratify patients by infarct size (e.g., ASPECTS 3–5 vs. 0–2) and mechanism of stroke (e.g., cardioembolic vs. atherothrombotic) to enable subgroup analysis. Timing from symptom onset to reperfusion should be tightly controlled and recorded, as delays may disproportionately harm large core patients. Incorporating biomarkers of blood–brain barrier integrity, such as circulating metalloproteinase‐9 levels, could help identify patients at heightened hemorrhagic risk, enabling tailored IVT use. Finally, harmonizing thrombectomy device selection and procedural technique across trial centers will help isolate the effect of IVT itself on outcomes.

## 5 Conclusion

This systematic review and meta‐analysis suggests that bridging therapy with IVT prior to EVT in patients with large ischemic core infarcts does not confer significant advantages over EVT alone in terms of functional outcomes, safety, or recanalization success. Given the risk of hemorrhagic complications associated with IVT, cautious patient selection remains essential. Future research, particularly RCTs, is necessary to establish definitive treatment guidelines for this high‐risk population.

## Author Contributions

MHEM: conceptualization and methodology. AE, MHEM, RMW, YA, ME, IS, IMA, ME, AE, AA, OY, YN, MA: investigation and data curation. MHEM, MA and IS: formal analysis. MHEM, IS, IMA and MA: Writing‐Original Draft. MHEM, IS and MA: Supervision. MHEM: Project administration. AE, MHEM, IMA, IS, MA: Writing‐Review & Editing. All authors read and approved the final content.

## Funding

The authors have nothing to report.

## Ethics Statement

The authors have nothing to report.

## Conflicts of Interest

The authors declare no conflict of interest

## Supporting information




**Supplementary Figures**: brb371052‐sup‐0001‐FigureS1‐S2.docx

## Data Availability

All data generated or analyzed during this study are included in this published article.
