# Supplementary material for: To Bridge or Not to Bridge? A Meta‐Analysis of Intravenous Thrombolysis Before Thrombectomy in Large Ischemic Core Strokes
Source: Brain Behav. 2025 Nov 21;15(11):e71052. doi: 10.1002/brb3.71052 (PMC12639189; doi:10.1002/brb3.71052)
Supplement: Supplementary file 1 — Supplementary Figures: brb371052‐sup‐0001‐FigureS1‐S2.docx [file BRB3-15-e71052-s001.docx]

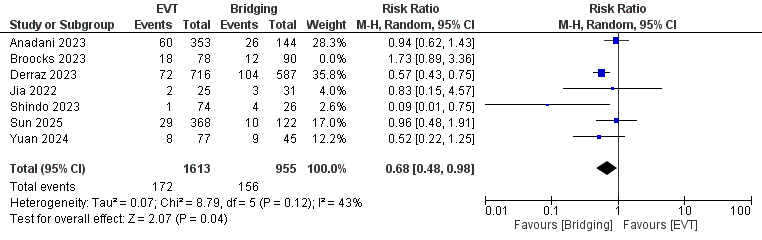


Supplementary figure 1: Sensitivity analysis by leave-one-out method for mRS 0-1 outcome


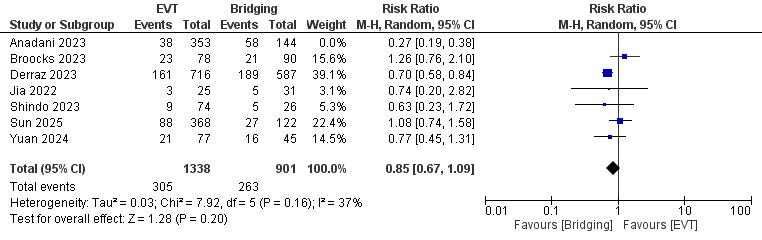


Supplementary figure 2: Sensitivity analysis by leave-one-out method for mRS 0-2 outcome
